# Protozoan Parasites in Drinking Water: A System Approach for Improved Water, Sanitation and Hygiene in Developing Countries

**DOI:** 10.3390/ijerph15030495

**Published:** 2018-03-12

**Authors:** Alua Omarova, Kamshat Tussupova, Ronny Berndtsson, Marat Kalishev, Kulyash Sharapatova

**Affiliations:** 1 Department of Nutrition and General Hygiene, Karaganda State Medical University, Gogol Street 40, Karaganda 100008, Kazakhstan; alua_1912@mail.ru (A.O.); marchelo99.62@mail.ru (M.K.); 2 Department of International Cooperation and Bologna Process, Karaganda State Medical University, Gogol Street 40, Karaganda 100008, Kazakhstan; 3 Division of Water Resources Engineering & Center for Middle Eastern Studies, Lund University, P.O. Box 118, SE-221 00 Lund, Sweden; ronny.berndtsson@tvrl.lth.se; 4 Department of Surgery, Gynecology and Pediatry, Semey State Medical University, Pavlodar Branch, Toraigyrov Street 72/1, Pavlodar 140000, Kazakhstan; sharapatova_kulyash@mail.ru

**Keywords:** protozoan parasites, drinking water, WASH, *Giardia*, *Cryptosporidium*, developing countries

## Abstract

Improved water, sanitation and hygiene (WASH) are significant in preventing diarrhea morbidity and mortality caused by protozoa in low- and middle-income countries. Due to the intimate and complex relationships between the different WASH components, it is often necessary to improve not just one but all of these components to have sustainable results. The objective of this paper was to review the current state of WASH-related health problems caused by parasitic protozoa by: giving an overview and classification of protozoa and their effect on people’s health, discussing different ways to improve accessibility to safe drinking water, sanitation services and personal hygiene behavior; and suggesting an institutional approach to ensure improved WASH. The findings indicate that *Giardia* and *Cryptosporidium* are more often identified during waterborne or water-washed outbreaks and they are less sensitive than most of the bacteria and viruses to conventional drinking water and wastewater treatment methods. There are various institutions of control and prevention of water-related diseases caused by protozoa in developed countries. Unfortunately, the developing regions do not have comparable systems. Consequently, the institutional and systems approach to WASH is necessary in these countries.

## 1. Introduction

Improved water, sanitation and hygiene (WASH) could prevent the deaths of more than two million children under the age of five annually [1]. The main cause of the mortalities is diarrhea [2]. African and South-East Asia regions accounts for 78% (1.46 million) of all deaths due to this disease in the developing countries [3]. Protozoan parasites are identified as the second most frequent etiological causes of the mortality among children under five years old. Globally they are responsible for 1.7 billion cases of diarrhea, which leads to 842,000 deaths per year [4,5]. Parasitic diseases transmitted through water by protozoa cause epidemic and endemic diseases in both developed and developing countries [6]. However, the former have better hygiene conditions so parasitic protozoa are commonly not considered as a reason of these diseases [7]. The latter, where population consume both treated and untreated water, have the high rates of protozoan diseases in spite of having achieved their water treatment standards. Thus, it is necessary to take many measures to improve WASH in resource-poor settings for identification of these organisms in developing countries [8].

During recent years, there appears to be an increasing number of cases with outbreaks of waterborne or water-washed parasitic diseases worldwide [9,10]. The reason for this seems to be crumbling or poorly maintained community sanitary and water supply systems [10,11]. In addition, tests for protozoa are not frequently performed, and absent in small treatment facilities. In practice, challenges in research of drinking water for cysts and oocysts of pathogenic enteric protozoa are withdrawal and delivery of significant quantities of water (50 L) to a lab, the duration of filtration, low probability of identification of cysts and oocysts and expensiveness of the method. That is why microbiological indices of water quality in Sanitary Rules usually include only indicator bacteria, especially in developing regions.

It is often difficult to distinguish the direct cause of diarrheal infection due to the complex interrelationships between WASH. This may be one of the reasons why increases in water supply and sanitation service coverage sometimes does not reduce diarrhea [1]. Due to the intimate and complex relationships between the different WASH components, it is often necessary to improve not just one but all of these components to have sustainable results.

The objective of this paper is to review the current state of WASH-related health problems caused by parasitic protozoa. We review the present treatment techniques for drinking water supply as well as sanitation and institutional management approaches to decrease risk for disease outbreaks. The focus is mainly on developing countries. To achieve the objective, we first give an overview and classification of protozoa and their effect on people’s health. Then we discuss different ways to improve accessibility to safe drinking water, sanitation services and personal hygiene behavior. Finally, we suggest an institutional and systems approach to ensure improved WASH.

## 2. Protozoa Classification and Occurrence

**Figure 1:**
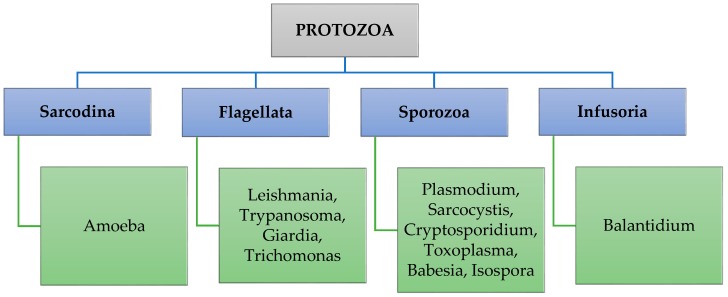
Classification of parasitic protozoa.

There are about 15,000 species of protozoa [12] on Earth. However, mainly the following classes have a significance for health: Sarcodina, Flagellata, Sporozoa, and Infusoria (Figure 1). Parasitic protozoa that are transmitted through water and those that cause human infections are *Toxoplasma gondii*, *Entamoeba histolytica*, *Cyclospora cayetanensis*, *Isospora belli*, *Blastocystis hominis*, *Balantidium coli, Acanthamoeba* spp., *Sarcocystis* spp. and *Naegleria* spp. However, the most common water-related parasitic infections are cryptosporidiosis and giardiasis [13,14]. *Giardia* and *Cryptosporidium* are zoonotic agents that are more often identified during outbreaks caused by contaminated drinking water. The majority of giardiasis outbreaks (71%) occurs in systems with surface water, while the majority of cryptosporidiosis outbreaks (53%) ensues in the groundwater system [6]. These enteric protozoan parasites are important causes of diarrheal disease [5,9,11,15], especially among children in developing countries [16].

Cysts and oocysts of protozoa are found in waste, surface, and groundwater sources, as well as drinking water samples even after treatment using conventional methods [6]. Since they mainly aim at the removal of pathogenic bacteria such as *Vibrio cholerae*, *Salmonella typhi*, *Salmonella paratyphi*, and *Escherichia coli* [17,18], chlorine-resistant parasitic protozoa such as *Cryptosporidium parvum* and *Giardia lamblia* are of particular concern [19,20,21]. Cysts of *Giardia* and oocysts of *Cryptosporidium* can penetrate through the water treatment system because of their small size (1–17 µm) and may cause outbreaks and epidemics after consumption of purified drinking water [22].

**Figure 2:**
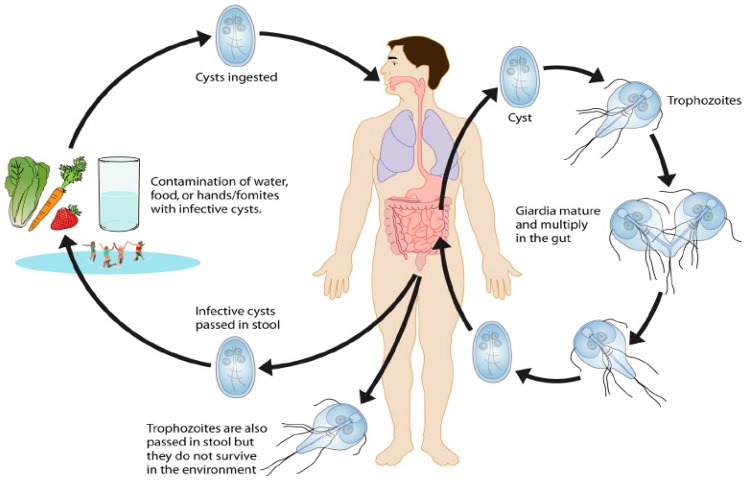
Life cycle of *Giardia* [24].

Figure 2 describes the life cycle of parasitic protozoa in terms of *Giardia* [23,24]. Cysts are responsible for transmission of giardiasis and oocysts for cryptosporidiosis. Both cysts and oocysts are resistant forms and can survive in cold water for several months. The infection begins with ingestion of cysts or oocysts in contaminated water, food, by hands or fomites into the digestive tract of the host. Each cyst produces two trophozoites in the small intestine. Trophozoites multiply by longitudinal binary fission, remaining in the lumen of the proximal small bowel where they can be free or attached to the mucosa by a ventral sucking disk. Encystation occurs as the parasites transit toward the colon. Both cysts and trophozoites can be found in the feces [23]. Because of defecation, cysts and oocysts are excreted into the external environment and infect other hosts [23,25,26].

## 3. Water Pollution Mechanisms

The source of infection for cryptosporidiosis and giardiasis is an infected human or animal, who secretes invasive cysts and oocysts in their feces. The transmission of *Cryptosporidium* and *Giardia* is fecal-oral. Infection occurs through drinking water or swallowing water while swimming in open pools [27]. The small size of protozoa allows them to pass through filters at drinking water treatment facilities. A study carried out in Japan showed that *Cryptosporidium* oocysts were detected in 35% (9/26) of filtered water samples (geometric mean concentration was 1.2 oocysts/1000 L) and *Giardia* cysts in 12% (3/26; geometric mean concentration was 0.8 cysts/1000 L) [28]. In addition, they have high stability in water and they are able to maintain viability up to 6–12 months or more in the aquatic environment. This is because of the fact that *Cryptosporidium* oocysts and *Giardia* cysts have a thick wall around them. The formation of such protective wall contributes to “freezing” of metabolism of protozoa, and they stay in the so-called “suspended animation” [29,30].

The main cause of waterborne and water-washed diseases is fecal material in the water supply and lack of hygiene [11]. Feces can enter the water in various ways such as [31]: wastewater overflow, nonfunctioning sewage systems, contaminated storm drains, and agricultural effluent. Causative agents of protozoan disease along with liquid sewage from improperly-arranged toilets, cesspools, and livestock farms penetrate into the soil and aquifers. Untreated livestock wastes from facilities located in close proximity to settlements that use the upper aquifers for water supply are especially dangerous. Melt and rain water on the ground can penetrate the groundwater aquifers and pollute the quality of water used for drinking. Confined water constitutes an underground reservoir between the confining strata with a time-constant level and relatively high-water quality [32]. Confined water is the most reliable in sanitary and parasitological terms. However, cysts and oocysts seeding even of confined water can occur if the integrity of the confining strata is violated or there is no supervision over old wells [31].

As mentioned above, statistics on direct causes for diarrhea outbreaks are difficult to assess due to the great uncertainty on interactive WASH components. The majority of laboratory-confirmed cases comes from the developed countries. For instance, in the USA, 411,041 cases of outbreaks caused by *Cryptosporidium* and *Giardia* associated with drinking water were registered for 1990–2012 [33]. According to these data, treatment deficiency was the most common cause during outbreaks. Obviously, number of outbreaks of waterborne or water-washed parasitic protozoan diseases in low- and middle-income countries are significantly higher. Unfortunately, we do not have comparable findings for the developing regions.

## 4. Drinking Water Treatment

Traditional methods of drinking water treatment include a number of processes. When applied to raw water sources, they contribute to reducing microorganisms that cause concern for public health [34]. Coagulation, flocculation, and settling act to separate solids from the liquid phase when particles settle under gravity. Microbial agents (protozoa, bacteria, and viruses) tend to sorb into coagulation or flocculation and thus removed. The *Cryptosporidium* oocysts and *Giardia* cysts removal efficiency is about 90% [6,35]. Unfortunately, these methods are regarded as economically unprofitable for developing countries. Plummer et al. [36] investigated the efficiency of clarification (sedimentation versus dissolved-air flotation) for removal of *Cryptosporidium* oocysts under various conditions. The results of this study showed that oocyst absorption was maximal at pH 5.0, and when coagulants were used at doses higher than those currently in use to remove turbidity [34,37].

With the proper design and operation, filtration can serve as a consistent and effective barrier for microbial pathogens [38]. The transfer efficiency depends on technological parameters such as the size and density of microbes, size and surface charge of organisms and coagulant particles, as well as depth of the filter material and filtration rate [39]. Rapid filtration, such as a simple screen filter, does not remove microbial pathogens effectively. Slow sand filters can be very effective in removing microbial contamination from water [6]. However, it has been shown that diatom filtration is more effective in reducing the concentration of *Cryptosporidium* oocysts and *Giardia* cysts than other conventional filtrations or granulated media [34,40,41,42].

Pressure-actuated membrane processes (microfiltration, ultrafiltration, nanofiltration, and reverse osmosis) play a significant role in the production of drinking water in the USA and Europe [43,44]. Microfiltration, ultrafiltration, nanofiltration and reverse osmosis have received great attention as an alternative to the traditional purification and removal of protozoal cysts [34,43]. Microfiltration membranes have the largest pores in the range from 0.1 to 10 µm and the highest permeability. Microfiltration is an effective process for removing particles that can cause problems in further processing. The use of microfiltration membranes in water purification includes clarification, pre-treatment, and removal of particles and microbes [34,43,44]. Ultrafiltration membranes have smaller pores (0.002–0.1 µm), so their permeability is much lower than in microfiltration, and high pressure is required. At present, the use of ultrafiltration membranes in water treatment involves the removal of particles and microbes. Physical sieving is considered to be the main mechanism for removal of protozoal cysts. The pore sizes for microfiltration and ultrafiltration used in water purification range from 0.01 to 0.5 µm, which is at least one order of magnitude smaller than the size of protozoan cysts (4–15 µm) [34,43]. Nanofiltration membranes have pores of around 0.001 µm. Nanofiltration withdraw divalent ions from water; thus, it is widely used for water softening. Reverse osmosis membranes have the smallest pores of approximately 0.0001 µm. Reverse osmosis is needed for drinking water preparation from seawater, brackish water or groundwater due to their ability to monovalent ions removal [45].

LeChevallier et al. [46] conducted a study of 66 conventional water systems in the USA and found that compliance with the criteria set out in the Surface Water Treatment Rule (SWTR) did not ensure that filtered water was free of water-related protozoa. Therefore, high levels of disinfection or more effective disinfection procedures were necessary to protect humans against water-borne protozoa such as *Cryptosporidium* and *Giardia*. Disinfection is an essential component for most treatment facilities, especially for those that use surface water, since granular filter material by itself will not remove most pathogens from water. The main reason for widespread occurrence of reagent methods of drinking water disinfection is the ease of continual assessment of their effectiveness. With a centralized water supply system, water quality monitoring based on epidemiological indicators must be carried out at least once per hour. In reagent disinfection methods, evaluation of the effectiveness of water disinfection is carried out by determining the residual amounts of disinfecting agent in it [34]. The main factors that influence the effectiveness of disinfection are concentration of the disinfectant, contact time, temperature, and pH (depending on the disinfectant) [6].

Chlorine is the most commonly used disinfectant for drinking water treatment in many developed and developing countries. The use of chlorine has a long history in water treatment [47], and it has been successfully used for both drinking water and wastewater. In addition, chlorine is recommended as a water treatment method in the household, especially in developing countries, since it is affordable and easy-to-use disinfectant [48,49]. Additional chlorine disinfection at point-of-use can reduce the risk of diarrhea caused by *Escherichia coli* among children by 29% [48]. Nevertheless, it has several disadvantages: ineffectiveness against protozoa, loss of effectiveness, strong odor and disagreeable taste due to organic material in the treated water [48,50]. In terms of resistance to chlorine inactivation, viruses and bacteriophages are considered to be more resistant than vegetative bacterial cells [47]. Jarroll et al. [51] determined that *Giardia* cysts had been relatively resistant to chlorine inactivation. *Cryptosporidium* is one of the most resistant microorganisms in the water. According to the published data, the inactivation of *Cryptosporidium* has not been observed even after 18-h contact with 1.05% and 3% chlorine [34,52,53,54]. Whereas chloroamination of drinking water has won popularity because of concerns about the hypothetical risks of long-term consumption of chlorinated by-products of disinfection. Monochloramine is considered to be a weak biocide compared to free chlorine, since it requires 25–100 times more exposure time than chlorine to achieve comparable inactivation [6].

The primary focus of many researchers is currently on alternative disinfectants including chlorine dioxide, ozone, and UV radiation [55]. Chlorine dioxide exists as an undissociated gas dissolved in water in the pH range 6.0–9.0. It is a strong disinfectant and, as a rule, it is considered that its biocidal efficacy is comparable to or slightly higher than that of chlorine under certain conditions [6,47]. Chlorine dioxide is an effective disinfectant against *Giardia* and *Cryptosporidium* (about 90% inactivation of cysts and oocysts). Nevertheless, this disinfectant form by-products like chlorite and chlorate. In addition, chlorine dioxide is about five to ten times more expensive than chlorine [56].

By comparison, ozone is a very strong oxidant, which is toxic to most pathogens in water, even for some protozoan cysts, such as *Cryptosporidium*. It is used to improve taste and color, as well as to remove organic and inorganic compounds in water [57]. Despite the advantages of ozone, it has a number of disadvantages that limit its use in water treatment such as high cost, need for operating and service infrastructure, and no residual protection in the distribution system [58].

A different view is ultraviolet water-disinfection occurs via the ability of UV radiation to penetrate the cell wall and reach its information center, i.e., nucleic acids DNA and RNA. Ultraviolet water-disinfection irreparably damages the DNA, which leads to impairment of cell replication and/or cell death [59]. The advantages of UV radiation are mainly that it does not depend on the use of chemical additives, is effective in inactivation of protozoan parasites, requires a relatively short contact time, and there are no disinfection by-products identified [34]. Its disadvantages, however, include differences in efficiency between various types of UV lamps and reactor designs, inability to measure the lamp dose in practice, interference of turbidity, and no residual protection in water distribution system [34].

Unfortunately, neither ozonization, nor ultraviolet radiation have bactericidal after-effect, therefore they may not be used as independent means of water disinfection when treating water for utilities, drinking water supply, or swimming pools. Ozonization and ultraviolet disinfection are used as additional methods of disinfection. When used in combination with chlorination, they increase its efficiency and reduce the number of chlorine-containing reagents added [34].

## 5. Sanitation Management

In industrialized countries, the use of treated wastewater for domestic, industrial and agricultural purposes is currently the most important method of reuse of wastewater when providing sanitary and environmental guarantees [60]. Wastewater treatment plants can become a source of pollution for drainage areas if the wastewater is not treated properly before being discharged to nearby rivers or ponds [19]. Moreover, cysts and oocysts can withstand conventional water disinfection (see previous section), so they can be found in significant amounts of treated wastewater [61,62,63,64,65].

Various studies of treatment plants, where only primary treatment was carried out or each treatment was considered individually, revealed low rates of removal efficiency in the primary stages [66,67]. The primary treatment includes elimination of contaminants, such as fats, oils, sand, gravel, and stones, which are easily collected and removed. The main goal of the primary stage is to obtain a homogeneous liquid that can be biologically processed. However, some treatment plants use only primary processing, and since the removal of parasites is not the goal of primary treatment, the efficiency of such facilities is minimal [60]. *Giardia* cysts and *Cryptosporidium* oocysts have not been completely eliminated even after secondary treatment. The study conducted in Spain by Castro-Hermida et al. [60] with an analysis of wastewater samples from 12 treatment plants showed that cysts and oocysts were presented in all samples of treated wastewater (100%) that flowed out of the treatment plants throughout the year, and the largest number of cysts and oocysts was found in spring and summer. The average removal efficiency for these parasites, which had both primary and secondary treatment processes, was 16% to 86% for *Cryptosporidium* spp. and 2% to 90% for *Giardia lamblia.*

Proper management, treatment, and dispersion of human and animal feces are important for hygiene and safety of drinking and recreational water. When the amount of feces entering the environment is taken into account, the magnitude of the problem may seem overwhelming [61,62,63,64,65]. Throughout the world, there is a big difference in the coverage of toilets. Approximately 1.77 billion people around the world use pit latrines as the primary means of sanitation.

Pit latrines are the simplest and most inexpensive form of improved sanitation. They usually consist of a round, rectangular or square hole in the ground and are covered with a concrete slab or a floor with a hole through which feces fall [68]. Pit latrines usually lack a physical barrier, such as concrete, between stored excrement and soil and/or groundwater [69]. In some countries where pit latrines are common, greater than two billion people use groundwater as a source of drinking water [68]. Therefore, contaminants from pit latrines can enter groundwater and create a threat to human health. The degree of transfer of microbes from the pit latrines to groundwater mainly depends on the ecological context of the area, especially hydrological and soil conditions. The distances and rates of movement of contaminants are determined by regulated by soil type, flow rate and direction of groundwater for natural and anthropogenic conditions, and biogeochemical conditions of groundwater [68]. The potential for large-scale contamination of groundwater by pit latrines also depends on social factors, such as use of toilets, toilet density, maintenance, and pumping of groundwater [68].

There are concerns that pit latrines can affect human health and the environment due to more common use of both pit latrines and groundwater resources in low-income countries. One gram of fresh feces from an infected person can contain about 10^6^ viral pathogens, 10^6^–10^8^ bacterial pathogens, 10^4^ protozoan cysts and oocysts and 10–10^4^ helminth eggs [70]. The toilet type, design, materials and quality of construction also affect the localization of contamination from pit latrines. Thus, it is necessary to take into account both ecological and anthropogenic factors to assess safety of location of pit latrines and groundwater sources [68]. Dzwairo et al. [71] stressed three important factors. Firstly, analysis of some critical parameters, such as the depth of infiltration layer and the direction of groundwater flow. Secondly, developing alternative sanitation, such as raised or leveled pit latrines, to minimize the impact on groundwater. Finally, application of a comprehensive approach including geotechnology and hydrogeology to solve sanitation problems.

Each country has its own standards for the construction of toilets. For instance, in Haiti, toilets should be located at least 30 m away from any source of surface or drinking water, and the excavation bottom should be at least 1.5 m above the maximum height of the groundwater level [72]. According to the recommendations for groundwater in South Africa, pit latrines should be located at least 75 m away from water sources [73]. WHO suggests a minimum risk of groundwater contamination if the distance between the pit latrine and groundwater level is greater than 2 m, provided that the fill rate is less than 50 L/m^2^/day. In addition, 15 m is proposed as a safe lateral distance between pit latrines and wells [74]. However, in later and more conservative recommendations, WaterAid [75] suggests that toilets and water sources should be at least 50 m apart. The Sphere project [76] recommended 30 m as the minimum standard for the lateral distance between local sanitation systems and water supply sources.

Pit latrines remain an important strategy for improving the conditions of human excrement removal despite the potential for groundwater contamination. This system is the most basic option for low-income countries to reduce the level of open defecation and expand access to improved sanitation. Given that approximately 1.11 billion people do not currently have sanitation [68], it is expected that pit latrines coverage will increase as people try to proceed from open defecation to basic sanitation [77]. Therefore, great efforts are needed to develop more reliable but viable approaches to the placement of pit latrines and water sources. The proposed guidelines must ensure the protection of groundwater from the entry of any pathogens [68].

## 6. Personal Hygiene

The main factors that reduce the relevance and impact of protozoal infections in the field of public health are education in sanitation and hygiene, abundant availability of good quality water, good sanitary conditions and adequate disposal of human and animal excrements [58]. Education and motivation to change people hygienic behavior should take place in the context of the family [78]. People can protect themselves and others from water-related protozoan diseases by practicing good personal hygiene, which includes washing their hands before preparing and eating food, after going to the bathroom, after changing diapers, and before and after tending to someone who is sick [79].

Even if there is an uninterrupted supply of microbiologically safe water, it can be contaminated by consumers at the household level through the improper use [80,81]. Therefore, the water tanks must be clean and closed, it is necessary to clean and disinfect them on a regular basis. When collecting or storing water, it is not allowed for anyone to put one’s hands into the water and drink directly from the water tank. If possible, water tanks should have a narrow neck and a stopper to avoid contact of water with hands, otherwise water must be taken from the tank with a ladle or a mug. In addition, it is necessary to use the available water to the end, and then rinse the tank thoroughly with clean water before next filling. Moreover, water for domestic purposes should be kept in tanks for as short a time as possible [82]. With non-centralized water supply, people should be aware of how important it is to protect the source of water supply from contamination by pathogenic protozoa, and how to do so, as well as take responsibility for the safety of water they consume. People should keep wells closed when installing a hand pump and proper drainage, also keep jugs, jars and other utensils, which are used to collect and store water, clean and in clean places. It is critical to dispose of feces and sewage away from any sources of water supply and build toilets according to the requirements. Finally, population should conduct a periodic sanitary inspection of water sources and water quality [82,83].

Water purification at a household level is another significant aspect of hygiene in developing countries. Some small water purification devices have been developed to be used directly on-site. For instance, filters can purify small water volumes at a household level. In this case, all filters have one common property, they must be operated in the correct manner (i.e., they must be regularly cleaned and maintained) [79]. However not all home water filters can remove parasitic protozoa. Therefore, it is believed that boiling is the best method of obtaining water that is free from biological contamination. In many developing countries, people routinely boil drinking water, as there is no confidence in the safety of water supply or it is under threat [84]. To kill or inactivate *Cryptosporidium* and *Giardia*, water should be kept at a rolling boil for one minute (at elevations above 6500 feet, boil for three minutes). Water should then be allowed to cool, stored in a clean sanitized container with a tight cover, and refrigerated [31,85]. Nevertheless, it is economically unprofitable and environmentally unsustainable to recommend daily boiling of drinking water to the population of developing countries with a low income. Therefore, boiling as a method of disinfection of drinking water can be recommended only in emergency situations and is used regularly only by those who can afford it [84].

Safe disposal of human feces, so that it is isolated from contact with humans, animals or flies, is the main barrier to prevent the spread of protozoan parasites contained in feces in the home and near-home environment [86]. According to Fewtrell et al. [87], improved conditions for the disposal of human feces can reduce the risk of intestinal diseases by 32%. The toilet should be flushed after each use, regularly brushed using special cleansers and flushed after that. It is also necessary to use a de-rusting solution in the toilet. The surface of the toilet bowl, as well as other surfaces that come into contact with hands, such as the edge, toilet seat, lid and flush handle must be regularly washed using special detergents, and it is significant to use a separate rag to wash the toilet bowl. Then, the brush and rag must be washed with soap or detergent, rinsed and dried well. In addition, the toilet must be kept closed to prevent the spread of infection by flies [82].

In the areas with no centralized sewerage, the disposal and treatment of human feces is done at the “local” level (septic tanks, pit latrine, etc.). It is important that the soil where the excrement seeps is properly selected and the toilet is correctly installed, taking into account the distance from the water supply sources and the depth of groundwater, and maintained well (see recommendations in the previous section). People who do not have access to basic sanitation should immediately bury their excrements and not defecate near the house [86]. Moreover, it is important to use suitable and safe methods of anus care. Even a small amount of remaining feces can contain a large number of pathogenic protozoa. The best way to care for the anus after defecation is to use toilet paper or other material, after which it should be discarded into the toilet bowl or dug into the ground. In many developing countries, after defecation people use leaves, sticks, stones, etc. In such cases, it is important that these items are safely disposed, for example, buried in the ground. If running water is used, it is necessary to rinse the anus thoroughly, flush, and then make sure that this water is not left on the toilet bowl or on the floor. Washing hands after defecation is also strictly recommended [82].

Since cryptosporidiosis and giardiasis are widespread among children, the safe disposal of children’s excrements is important in preventing the transmission of these protozoan diseases [9,88]. Small children should be taught to use a pot. It is required to use toilet paper to wipe children’s bottoms, and if it is not available, for example, in rural areas, to rinse the bottom under running water or with water from a bucket, so that the rinse water accumulates in the pot. This is important, since the rinse water can contain pathogenic protozoa. The contents of the pot, toilet paper, etc. must be poured out into the toilet bowl. The pot must be washed, dried out and covered so as not to attract flies. If children defecate without a pot, the feces must be disposed of immediately by pouring it into the toilet bowl or digging into the ground. If defecation occurs in the house, the place needs to be washed and, if possible, disinfected. It is also necessary to wash hands after such hygienic procedures with a child [89]. Consequently, improved personal hygiene behavior is a crucial factor in the prevention of cryptosporidiosis and giardiasis.

## 7. Institutional Approach to Ensure Improved WASH

The provision of clean water and sanitation for all is one of the Sustainable Development Goals (SDG), otherwise known as the Global Goals [90]. SDG 6 comprises six technical targets for the period until 2030 relating to drinking water, sanitation and hygiene, wastewater management, water efficiency, integrated water resource management and protection of aquatic ecosystems [91]. We should mention the first three of them. Target 6.1 of SDG is to achieve universal and equitable access to safe and affordable drinking water for all, which is located on the premises, available when needed and free of fecal and harmful chemical contamination. Target 6.2 is to achieve access to adequate and equitable sanitation and hygiene for all and end open defecation, paying special attention to the needs of women and girls and those in vulnerable situations. Access to safely managed sanitation is crucial and implies a basic sanitation facility, which is not shared with other households and where excreta are safely disposed in situ or treated off-site. Target 6.3 is to improve water quality by reducing pollution, eliminating dumping and minimizing release of hazardous chemicals and materials, halving the proportion of untreated wastewater and substantially increasing recycling and safe reuse globally [91]. Universal SDG targets can only be considered achieved when met for all sub-groups within the population, which implies progressive disaggregation of data by income, gender, age, race, ethnicity, migratory status, disability, geographic location and other characteristics relevant in national contexts.

**Figure 3:**
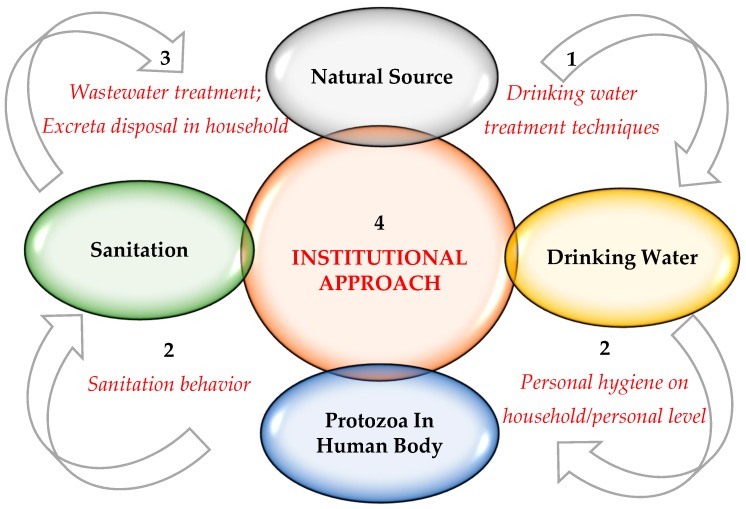
Schematic of systems approach to ensure improved water, sanitation and hygiene (WASH).

In order to achieve these targets regarding drinking water sources free from protozoan parasites, the institutional and systems approach should be used such as described in the following figure (Figure 3):

1. Access to safely managed drinking water source;
2. Improved personal hygiene behavior;
3. Access to safely managed sanitation services; and
4. Institutional approach to ensure the efficient implementation of measures to accomplish the above targets measures.

Some parts of the schematic can overlap with water safety plans (WSPs) recommended by the World Health Organization. WSPs encompasses the water supply from catchment to consumer and have three components, which are the responsibility of suppliers: a system assessment, effective operational monitoring, and management and communication [92]. However, the penetration of parasitic protozoa into the human body is heavily dependent on people’s behavior, which should be targeted at the prevention of the ingestion of protozoa with water and the transfer of cysts and oocysts into water sources. Therefore, the division of responsibility between monitoring institutions and consumers is essential for the implementation of these plans.

The USA has set up some organizations like the Center for Disease Control and Prevention (CDC) and the US Environmental Protection Agency (USEPA), which have monitored the outbreaks of water-related diseases since 1971. Sweden and Japan (in 1980 and 1981, respectively) established the system of National Epidemiological Surveillance of Infection Diseases (NESID) [93]. The National Notifiable Diseases Surveillance System (NNDSS) was created in Australia in 1990. The Health Protection Agency (HPA) was founded in the United Kingdom in 2003. The Public Health Agency of Canada (PHAC) was established in 2004. Following the USA, in 2005 some European countries organized the European Centre for Disease Prevention and Control (ECDC) [5]. These organizations monitor and evaluate the quality of environmental medium including water sources due to their impact on public health and develop recommended practices for reducing any adverse impacts. Monitoring of the environmental condition and public health is carried out at the government level. One of the most significant aspects of this activity is the study and analysis of parasitic morbidity. Numerous departments and research laboratories are used to collect and analyze information [93]. Relevant information and documentation on outbreaks caused by parasitic protozoa are available from most of these centers [5].

In 1994, USEPA published the *Cryptosporidium* Criteria Document [94] and thus declared *Cryptosporidium* to be the main pollutant of drinking water. The attention to this genus of protozoa has increased and subsequently led to further investigation. Detection of *Cryptosporidium* and *Giardia* in water requires specialized equipment, skills in order to provide reliable data that can be used to monitor compliance, and to determine the microbiological risks of diseases associated with drinking water [34,95]. USEPA has approved Method 1623 for simultaneous detection of *Cryptosporidium* and *Giardia*. This method is considered the gold standard and requires filtration, immune-magnetic separation of cysts and oocysts, immunofluorescence analysis to determine protozoan concentrations with confirmation via staining with live dyes (4.6-diamidinophenylindole) and microscopy of differential interference contrast [96]. Any alternative procedures are allowed provided that the required quality tests are carried out and all quality control criteria in these methods are met. Although, the Method 1623 is now available for monitoring of *Cryptosporidium* and *Giardia* in water, other powerful methods include molecular analyses, such as polymerase chain reaction (PCR) alone or in combination with an analysis of infectious cell culture (cell culture-PCR). These methods are used for genetic typing as well as to determine the viability/infectivity and genotypes of *Giardia* cysts and *Cryptosporidium* oocysts [97,98,99,100,101,102,103]. Such tools should be used in studies to gain further understanding of transmission of these pathogens. The problem is that these methods are not affordable for the low- and middle-income countries [96].

Most developing countries do not have any governmental systems for recording the incidence and prevalence of water-related protozoal infections or outbreaks. Therefore, there is a lack of documentation on parasitic focal points of water-related diseases [5] which may have led to underestimated rates of protozoal infections in developing countries. About 600 million people in Latin America, Asia, and Africa live under unsanitary conditions [6], 1.1 billion people lack access to improved sources of drinking water and 2.6 billion people have no adequate sanitation [104]. Therefore, a high prevalence of water-related parasitic diseases can be expected in developing countries. Most outbreaks of giardiasis occur in Latin America, Africa, and Asia, about 5100 new cases each year [105]. The prevalence of *Cryptosporidium* in fecal samples from patients with gastroenteritis is 1–4% in Europe and North America, while the figure in Africa, Asia, Australia, and South America ranges from 3% to 20% [106]. Also, there are high rates of asymptomatic carriage of *Cryptosporidium* (10–30%) in developing countries, as compared to developed ones (<1%). Higher prevalence rates in developing countries can also be estimated for other water-related protozoal infections. However, most studies on the prevalence of parasitic infections are carried out in developed countries, where the health infrastructure and laboratory tests are more affordable than in the developing countries [5,107].

Although developed countries have established epidemiological surveillance systems, there is still no international agreement on the reporting structure. Hormann et al. [108] criticized the fact that the surveillance and reporting systems vary widely between different countries and comparison of data is not always possible. Although the CDC records each waterborne outbreak by agent, location and number of cases, European epidemiological surveillance systems are used to determine national infection rates and incidents, thus neglecting the details of water-related outbreaks. In addition, Craun et al. [109] found that these epidemiological surveillance systems often failed to identify the cause and source of infection. Therefore, even those countries that already support the surveillance system for waterborne parasitic outbreaks should improve their methods of detecting and diagnosing diseases.

The introduction of epidemiological surveillance systems in developing countries will be useful in detecting and combating parasitic protozoa, which can help to improve the public health. Therefore, it is necessary to develop reliable and affordable diagnostic instruments to identify pathogenic protozoa, especially in developing countries. A further study of new methods is required in order to obtain more results on protozoan diseases. In addition, all countries should establish an international standard reporting system leading to standardized databases and a successful cooperation in the control of waterborne pathogenic protozoa [5].

## 8. Discussion

The objective of this literature review was to present an overview of the current state of WASH-related health problems caused by parasitic protozoa. Clean water, proper disposal of bio-waste and improved hygiene behavior are important factors in preventing the transmission of cryptosporidiosis and giardiasis [110]. Protection of drinking water from these protozoa is a serious problem for water supply organizations around the world. Despite the fact that great success has been achieved in the field of water purification, *Cryptosporidium* and *Giardia* remain the two most important water pathogens. Good understanding of the mechanisms is required for adequate control of these parasites and there is a need for new and innovative purification methods that can be used in both developing and developed countries. This can only be achieved through integrated studies that examine the sources, concentrations, survival and transmission of water-related parasites, the environmental exposure, and, finally, the ability of purification systems to reliably reduce the risk of transmitting the disease by protozoa through water [34].

The guiding principle for providing safe water is the concept of a multiple barrier, which includes protection of the water source (surface and groundwater), optimization of water purification processes, and proper maintenance of distribution systems. In addition, a multiple barrier approach should be applied in the process of water purification. The treatment of drinking water, which includes a combination of different disinfectants and filtration technologies to remove and inactivate various microbial pathogens, will guarantee a lower risk of microbial contamination [34].

Since protozoan parasites are known to live in the environment for many months [111,112], there is a need to study better purification methods that can more effectively eliminate these organisms to prevent further waterborne and water-washed outbreaks caused by *Giardia* and *Cryptosporidium* [113]. Nowadays there are no requirements for testing recreational waters for protozoan parasites, although it has been shown that these pathogens can be discharged into recreational water during outbreaks [114,115]. Unlike swimming pools, recreational beaches have bottom sediment that can contain bacterial and parasitic pollution indices 1000 times more than overlying water [116]. Thus, it is important to conduct parasitological control in treatment plants and to establish regulations for acceptable concentrations of cysts and oocysts based on the subsequent use of wastewater [60].

Furthermore, besides improving water supply and sewerage systems to prevent or minimize the risk of spreading protozoan parasites, measures should be focused on the hygienic behavior of people [110]. People should feel their share of the responsibility for health and undertake such activities as hand washing, boiling tap water or installing additional filters for water purification, safe disposal of human waste and health education.

## 9. Conclusions

In conclusion, the institutional and systems approach to WASH is necessary to solve health problems caused by parasitic protozoa. Firstly, proper drinking water purification is necessary to ensure access to safe drinking water. Secondly, personal hygiene on household and personal level is required to prevent the penetration of parasitic protozoa into the human body. Thirdly, good sanitation behavior is able to protect our body from reinfestations. Finally, proper wastewater treatment or excreta disposal in household is significant in order for protozoan parasites in feces do not enter the sources of water supply. An appropriate body should monitor this complex system. Consequently, water monitoring systems for parasitic protozoa are essential in developing countries. This monitoring will be used with a view to assess risks in order to determine the required treatment course, to assess the risk for population, reliability and effectiveness of a full-scale water treatment system as well as to assist in the investigation of water-related outbreaks and epidemics.
